# Update on the Role of Poly (ADP-Ribose) Polymerase Inhibitors in the DNA Repair-Deficient Pancreatic Cancers: A Narrative Review

**DOI:** 10.1089/pancan.2020.0010

**Published:** 2020-12-04

**Authors:** Anup Kasi, Mohammed Al-Jumayli, Robin Park, Joaquina Baranda, Weijing Sun

**Affiliations:** ^1^ Division of Medical Oncology, Department of Medicine, Kansas University Cancer Center, Kansas City, Kansas, USA.; ^2^ Department of Medicine, MetroWest Medical Center/Tufts University School of Medicine, Framingham, Massachusetts, USA.

**Keywords:** PARP inhibitor, pancreatic adenocarcinoma, DNA damage repair, combination therapy

## Abstract

**Purpose:** Pancreatic ductal adenocarcinoma (PDAC) is the most common cancer found in the pancreas. It has a dismal prognosis and current therapeutic options, including surgical resection, provide only a temporary or limited response due to the development of treatment resistance.

**Methods:** A narrative review of studies investigating poly (ADP-ribose) polymerase (PARP) pathway inhibitors in metastatic PDAC to highlight recent advances.

**Results:** Mutations in BRCA genes confer a higher risk of PDAC, while germ line mutations are found in 4–7% of individuals harboring pancreatic cancer. Although solid tumors with defective DNA damage repair defect (DDR) genes such as BRCA show heightened sensitivity to platinum agents, tumors can exploit the PARP pathway as salvage pathways. Therefore, blocking this pathway will trigger cell death in vulnerable tumor cells with BRCA/DNA repair deficiency. Several drugs with inhibitory activity on the PARP pathway have been approved for breast and ovarian tumors harboring germ line or somatic BRCA mutations. Based on these results, the phase III POLO study showed a significant improvement in progression-free survival compared with placebo in BRCA mutant pancreatic tumors and highlighted the importance of germ line testing in everyone diagnosed with pancreatic cancer. In addition, expansion of the PARP inhibitor indication beyond BRCA mutations to other genes involved in DDR such as ATM and PALB2 merits attention.

**Conclusion:** PARP inhibitors represent a safe and efficacious treatment for a subset of PDAC patients with BRCA mutations. Ongoing trials are evaluating PARP inhibitors in PDAC patients with non-BRCA DDR gene deficiencies as well as PARP inhibitors in combination with other agents, notably immune checkpoint inhibitors to expand the group of patients that derive benefit from this treatment.

## Introduction

Pancreatic ductal adenocarcinoma (PDAC) is the most commonly diagnosed type of pancreatic cancer with a 5-year survival less than 9% for metastatic disease.^1^ The only curative treatment is surgical resection, but only 20% of new cases are eligible. The remainder present with distant metastasis or locally advanced disease and although a minority are eligible for neoadjuvant therapy with surgical resection, for the majority, palliative intent systemic chemotherapy is the only option. The metastatic population has a dismal median survival of ∼6 months.^2^

Precision medicine, the concept of treatment individualized to the patient based on specific target genes, has gained a foothold in oncology, including in PDAC treatment.^3,4^ Several attempts have been made to augment chemotherapy by combining with agents that target the tumor and the tumor microenvironment, but have failed to produce clinically meaningful outcomes. For example, the tumor microenvironment (TME) of PDAC comprises various molecules, including specific types of hyaluronic acid (HA).

By metabolizing tumor-associated HA, PEGPH20 activity results in TME remodeling. However, a recent phase III trial demonstrated no improvement in clinical outcomes with PEGPH20 despite pre-clinical studies suggesting its association with increased drug delivery of chemotherapeutic agents and enhanced tumor response.^5,6^

However, recent studies have investigated poly (ADP-ribose) polymerases (PARPs) as potential therapeutic targets with some success. PARP enzymes are involved in various intracellular processes, including regulation of gene expression, cell division, maintenance of genomic integrity, and apoptosis. Among the known members of the PARP family enzymes, PARP1 and PARP2 are the most clearly implicated in DNA repair.

Furthermore, PARP1 among these two enzymes is the best characterized, most abundant, and ubiquitous.^7^ PARP1 has a major role in DNA single-strand break (SSB) identification and repair via base excision repair; consequently, its inhibition leads to DNA double-strand breaks (DSBs).^8^ Of note, PARP3 has also recently been implicated in SSB repair. Taken together, PARP1–3 are the target molecules of PARP inhibitors. Notably, the PARP inhibitors olaparib and rucaparib are specific for PARP1–3, whereas talazoparib, niraparib, and veliparib are PARP1–2 specific.

The early PARP inhibitors were nicotinamide analogues first synthesized more than 40 years ago. These agents were further developed into the more potent drugs such as olaparib that recently entered the clinical setting.^9^

Increasing the DNA DSB burden in cancer cells via PARP1 inhibition is crucial in the treatment of tumors harboring mutant breast cancer susceptibility genes 1 and 2 (*BRCA1* and *BRCA2*).^10^ They are tumor suppressors with involvement in multiple intracellular pathways, most important of which is the role of regulating the DNA damage repair defect (DDR).^11^

There are two major DNA damage repair pathways in the repair of ionizing radiation-induced damage such as those occurring in the clinical settings: the nonhomologous end-joining and homologous recombination (HR) pathways. While the latter is predominantly involved in actively proliferating cells (by targeting G2 and S), the former functions regardless of cell cycle stage.^12^ Although both *BRCA1*/2 are implicated in HR, each enzyme acts on different steps of the pathway where BRCA1 mediates signaling and BRCA2 recruits RAD51 to DSBs to initiate repair.^12^

The central mechanism by which PARP inhibition leads to cell death is synthetic lethality. Animal models with *PARP1* deficiency exhibit intact viability and fertility, even though cells feature defective DNA SSB repair, likely because of compensatory activity in error-free HR.^13^ On the contrary, homozygous *BRCA1/2* mutant breast tumor cells are extremely sensitive to PARP inhibitors.^13,14^ Taken together, these findings indicate that whereas the loss of a single gene or protein will be insufficient to induce cell death, the concomitant loss of two genes or proteins involved in separate cellular pathways, for example, PARP1 and BRCA, will result in inviable cells, illustrating the concept of synthetic lethality.^15^

Because BRCA1 directs the MRE11/RAD50/NBS1 complex to DSB sites, homozygous mutant BRCA1 cells have highly suppressed DNA DSB repair responses.^16^ Therefore, defective BRCA together with PARP1 inhibition, which increases DSB burden, results in synthetic lethality.

The clinical applicability of PARP inhibitors in promoting tumor cell death was observed in ovarian cancer. Olaparib (Lynparza) showed activity in individuals with ovarian tumors harboring germ line *BRCA1/2* mutations and was FDA approved in 2014 for use in ovarian cancer.

In March 2017, following a report from the NOVA trial, niraparib was approved for the maintenance therapy of patients with recurrent ovarian cancer who fulfill the common criteria for platinum sensitivity.^17^ It was the first FDA-approved PARP inhibitor to be used in the maintenance setting and has since changed the standard of practice. Similarly, the first PARP inhibitor to gain approval in breast cancer was olaparib (Lynparza), which was based on the results of the OlympiAD trial (phase III). In 2018, the FDA approved olaparib for metastatic breast cancer with negative *HER2* and germ line *BRCA1/2* mutations after progression on prior therapy.^18^

Unfortunately, however, the success of PARP inhibitors has not been replicated in BRCA mutant tumors originating in sites other than the breast and ovaries. To this end, ongoing investigations are hoping to identify cancer types with favorable responses to PARP inhibitors. Herein we discuss the biological importance of PARP and its application in clinical settings, as well as the direction of future research and development pertaining to PARP inhibitors in metastatic PDAC.

## PARP Inhibitor As a Single Agent

### BRCA germ line mutations

#### PARP inhibitor second-line trials

Among the 10–16% of PDAC cases with family history, only 5% have inherited syndromes such as Peutz–Jeghers syndrome, familial atypical multiple-mole melanoma, hereditary nonpolyposis colorectal cancer, and hereditary breast and ovarian cancer, which arise from germ line mutations in *STK11*, *p16*, *MLH1*, and *BRCA1/2*, as well as other mismatch repair genes.^19,20^ Moreover, a strong family history (multiple first-degree relatives affected) confers increased risk even without such germ line genetic mutations.

*BRCA1/2* and *PALB2* are the most commonly found genes underlying familial PDAC. *BRCA2* mutations confer a 3.5 times higher risk of PDAC and are responsible for a sizable subset of familial PDAC (up to 17%).^21,22^ Furthermore, the prevalence of sporadic germ line *BRCA* mutations is ∼4.5%; however, this estimate may be higher in specific ethnic populations such as the Ashkenazi Jews, in whom it may be up to 15%.^23^

In addition, the prevalence of BRCA mutations may be even higher when accounting for rare germ line variants. For example, one study of PDAC specimens identified *BRCA1/2* or *PALB2* variants in 31% (13/42) of specimens. Among the identified *BRCA* variants, one was a known pathogenic variant (*BRCA2*^S2148fs^), two were found to be enriched in tumor tissues (*BRCA2*^R18H^ and *BRCA2*^G2044V^), and two were novel variants (*BRCA2*^K799R^ and *BRCA2*^R2964T^). Indeed, these rare and novel germ line *BRCA* variants suggest the underestimation of *BRCA* prevalence and underutilization of clinically actionable genetic variants in the setting of PARP inhibition in PDACs.^24^

Furthermore, several ongoing clinical trials are evaluating various PARP inhibitors in PDAC and are enrolling patients with germ line or somatic *BRCA1/2* or *PALB2* mutations.^25,26^ One phase II basket trial for olaparib with germ line *BRCA1/2* mutant cancers (breast, ovarian, pancreatic, or prostate) with recurrence demonstrated a favorable objective response (OR) (26% [95% CI 21.3–31.6]) in the overall population and OR in pancreatic cancer (21.7% [95% CI 7.5–43.7]).^27^ Another phase II trial in metastatic PDAC harboring germ line *BRCA1/2* mutants also demonstrated favorable results (1/23 complete response, 4/23 partial response [PR], and 6/23 stable disease [SD]). A phase II study of veliparib also in *BRCA* mutant PDAC showed 1/16 unconfirmed PR and 4/16 SD, and a median progression-free survival (PFS) of 1.7 months.

To improve results, additional strategies such as combination therapies are being pursued.^28^ Similarly, the RUCAPANC trial (NCT02042378) enrolled 19 patients (16 germ line and 3 somatic *BRCA* mutations) with advanced or metastatic PC. which progressed on 1 or 2 lines of chemotherapy. Other phase II trials are evaluating niraparib as a single agent in PDAC with homologous recombination deficiency (HRD) (NCT03601923, NCT03553004). The OR was 15.8% (3/19) with disease control rate at week 12 of 31.6% (6/19) overall, and 44.4% (4/9) had progressed on prior chemotherapy. The study concluded that rucaparib as a single agent has a tolerable toxicity profile and may be efficacious in metastatic PDAC with *BRCA1/2* mutations.^29^ Taken together, these clinical trials suggest that germ line *BRCA* mutant PDAC is responsive to PARP inhibition.

### PARP inhibitor maintenance trials

Based on the success of the POLO trial, the FDA approved olaparib as maintenance therapy for confirmed or suspected germ line *BRCA* mutated advanced PDAC. The study enrolled 154 patients in total with germ line *BRCA*1/2 mutant PDAC with SD or PR to first-line systemic therapy. Patients in the olaparib arm achieved strikingly durable responses of longer than 2 years and the study met its primary end-point of PFS (7.4 months vs. 3.8 months in favor of olaparib; hazard ratio 0.53 [95% CI 0.35–0.82]). Moreover, as expected, the rate of adverse events and health-related quality of life was comparable between the comparative arms. However, preliminary analysis of median overall survival (OS) (at data maturity of 46%) demonstrated no significant difference (18.9 months vs. 18.1 months, olaparib and placebo, respectively; hazard ratio 0.91 [95% CI 0.56–1.46]).^30^

In addition, FDA approval was granted for *BRCA* analysis CDx (Myriad Genetics) as a companion diagnostic test to facilitate the identification of *BRCA* mutant PDAC patients who may be eligible for olaparib.^31^ Along with *BRCA1/2* deficiency, pancreatic cancers that harbor other DNA repair defects also show a trend toward improved OS when compared with DNA repair-proficient cancers. Treatment with first-line platinum-based chemotherapy favored better OS in DNA repair-deficient PDAC cancers.^32^ Treatment with platinum-based therapy may have survival benefit.^25,33^

### Role of PARP inhibitors beyond BRCA germ line mutation: BRCAness

A subset of metastatic PDAC patients harbor defects in DDR genes, which are genes involved in initiating or mediating DNA repair mechanisms. These are *BRCA1*, *BRCA2*, *PALB2*, *CDKN2A*, *ATM*, *CHEK2*, *APC*, and *MUYTH*.^34^ From 650 unselected patients with pancreatic neoplasms, Lowery et al. found that pathogenic germ line alterations associated with the DDR were present in 20% of patients.^35^

In addition to *BRCA1/2* and *PALB2*, which are central to HR, defects in other genes may lead to HRD, a defect in any of the genes involved in HR repair, along with sensitivity to DNA damaging drugs including PARP inhibitors. Altogether, somatic mutations in HRD genes occur in ∼3.9–35% of tumors and lead to a so-called BRCAness trait (phenotype occurring in carriers of *BRCA1/2* mutations as well as in some sporadic tumors).^36–38^

Of note, a recent meta-analysis showed no difference in the OR to PARP inhibition in solid tumors regardless of whether *BRCA* mutations were germ line or somatic in origin.^39^ The NOVA trial supported this hypothesis by showing that niraparib resulted in responses in ovarian cancer patients who have progressed on platinum agents.

Notably, the remarkable PFS in niraparib versus placebo (hazard ratio 0.38 [95% CI 0.24–0.59]) was seen in patients with non-*BRCA* HRD gene mutations.^17^ Indeed, genes other than *BRCA* such as those aforementioned may be involved in the HRD phenotype, which can be assessed indirectly via the composite HRD score that measures genomic instability.^17^ Consequently, the HRD score has been used in various settings as a biomarker for platinum and PARP inhibitor sensitivity. Furthermore, defective DDR pathways in PDAC not only represent exciting therapeutic targets, but may be a prognostic marker as well, demonstrated by Kasi et al., studying the role of DDR genes as prognostic biomarkers for predicting improved OS and PFS. Furthermore, the trial also evaluated the efficacy of niraparib in DDR-deficient pancreatic tumor patients.^40^

## Strategies Combining PARP Inhibitors with Other Treatment Modalities

Despite the promising results demonstrated in clinical trials, PARP inhibitors, like other targeted therapies, have short-lived responses; therefore, combination therapy and identification of synergistic therapies have been emerging as a potential strategy to overcome this important issue of therapeutic resistance.

### Combined with chemotherapy

Previous evidence suggests an association between sensitivity to platinum agents and PARP inhibitors, particularly in ovarian cancer.^41^ Further studies suggest synergy between these agents since pre-existing DNA damage enhanced PARP activity. On the contrary, HRD PDAC tumors such as those with somatic BRCA2 mutations or familial pancreatic cancer heighten platinum sensitivity.^42^ Furthermore, patients without platinum resistance appear to respond better to PARP inhibitors.

In the SWOG S1513 trial (NCT02890355), the combination of mFOLFIRI and veliparib was compared with FOLFIRI alone for metastatic PDAC in the second-line setting in biomarker nonselected patients. Of the 108 patients included in this analysis, almost 30% of patients harbored DDR deficiency, including HRD in 9%. However, the study concluded that veliparib lead to no improvement in OS while increasing toxicity when combined with the chemotherapeutic agent. These findings suggested that selection of patients based on BRCA1 or BRCA2 and DDR biomarkers in future studies evaluating PARP inhibitors in PDAC may be more relevant in teasing out patients standing to benefit from such combination therapies.^43^

In this regard, a phase I/II study (NCT01489865) found veliparib plus FOLFOX potentially efficacious with an acceptable toxicity profile in BRCA2 mutant metastatic PDAC. In this study, 22 patients received treatment with median age of 64 years and Eastern Cooperative Oncology Group, Performance Score of 1. Half of the patients were previously untreated and two patients harbored defective BRCA2 genes. Whereas the first 6 enrolled patients were affected by grade 2 myelotoxicity leading to protocol amendment, the 16 patients enrolled subsequently demonstrated acceptable toxicity with no dose-limiting toxicity. The subset of treatment-naive patients demonstrated promising efficacy (OR 18%; median PFS 4.3 months; median OS 7.7 months). Of note, the two patients with germ line BRCA mutations had a significant durable response of greater than 17 months.^44^

In an international phase II study recently presented in GI ASCO 2020, O'Reilly et al. studied the combination of cisplatin and gemcitabine with or without veliparib in PDAC patients with germ line *BRCA* or *PALB2* mutations. With a total number of 52 patients enrolled in the trial, patients were randomized in a 1:1 ratio. Arm A received gemcitabine, cisplatin, and veliparib, whereas arm B received only gemcitabine and cisplatin. The OR in the patients in the triplet arm was 74.1% compared with 65.2% in the patients receiving the gemcitabine/cisplatin doublet (*p* = 0.55).

However, no difference was seen in the median PFS (10.1 months [95% CI 6.7–11.5] with the triplet vs. 9.7 months [95% CI 4.2–13.6] with the doublet) nor in the median OS (15.5 months [95% CI 12.2–24.3] with the triplet and 16.4 months [95% CI 11.7–23.4] with the doublet).^45^ It is worth mentioning that the triplet combination came at the expense of more hematologic toxicity, with more anemia, thrombocytopenia, and neutropenia, and more dose reductions and delays.

Based on the excellent response rates in both arms and the higher rate of hematologic toxicity in the triplet arm, the study group concluded that gemcitabine plus cisplatin is an efficacious regimen in metastatic germ line *BRCA* or *PALB2* mutant PDAC and that the addition of veliparib offered no OR benefit and suggested gemcitabine and cisplatin as the standard therapy in this population.^46^

Other trials are exploring the combination of PARP inhibitors with nonplatinum agents. For instance, a phase I trial (NCT03337087) is evaluating irinotecan, liposome, fluorouracil, and leucovorin with rucaparib as second-line therapy in nonselected and *BRCA1/2* or *PALB2* selected metastatic PDAC. As toxicity is a concern with chemotherapy combination, sequencing chemotherapy and PARP inhibition is being explored as in an ongoing study.

The aforementioned POLO trial is the best successful example of this approach. The study demonstrated prolongation of PFS with olaparib maintenance therapy for subjects with germ line BRCA mutant-advanced PDAC, nonprogressed on platinum agents. Nonetheless, no OS benefit was seen, and PFS benefit was seen only in *BRCA* mutant patients. Therefore, the combination therapy of PARP inhibitors with other agents should be explored in tumors without *BRCA* mutations.^30^

### Combined with targeted therapy

Combination therapy of PARP inhibitors with other targeted therapies has been implemented with the aim to induce the HRD phenotype. The goal of these treatment combinations is mainly to enhance its activity by inhibiting DDR via tissue hypoxia or by inhibiting DNA damage surveillance mechanisms in the cell cycle. The results of several studies conducted in ovarian and breast cancer could be extrapolated in the future to help treat metastatic pancreatic cancer patients such as studies evaluating PI3K (NCT01623349, NCT02208375) and VEGFR signaling (NCT02345265), and cell cycle checkpoint protein inhibitors such as WEE1 (NCT02511795).^46–49^

Despite being one of the most common family of oncogenes, RAS genes have been a notoriously fickle and elusive therapeutic target. Importantly, mutations in KRAS are the initial mutational events for most PDACs (95%).^50^ Furthermore, pancreatic carcinogenesis has been found to be dependent on constitutively high KRAS activity and KRAS defects are central in the development and progression of pancreatic carcinogenesis.^51^

Prior pre-clinical studies had serendipitously discovered potential synergistic cytotoxicity of mitogen-activated protein kinase kinase (MEK) inhibitors with PARP inhibitors *in vitro* and *in vivo* across various mutant RAS animal models. Furthermore, this synergy is likely to have wide-ranging applicability since this combination is effective regardless of *BRCA1/2* and *p53* mutation status.^52^ Therefore, this particular combination therapy should be investigated in tumors with RAS mutations, in which effective treatments are limited.^52^

In addition, results from other studies indicate that pharmacological targeting of MEK results in HRD and consequently PARP inhibitor sensitization in BRCA2-proficient tumors.^53^ There is a strong mechanistic basis for the synergy seen in combined PARP and MEK inhibition in the setting of RAS mutations. These include suppression of HR DNA repair, inhibition of cell cycle DNA damage surveillance mechanisms, as well as enhancement of BIM pathway-mediated apoptosis.^52–54^ RAS pathway activation induces replication stress and RAS pathway activation increases HR RAS pathway. This activation is indicative of PARP resistance and PARP-resistant cells acquire RAS mutations and increased signaling. Therefore, inhibiting MEK or ERK increases PARP activity in RAS-mutant or PARP-resistant cell lines. The underlying mechanism may be dependent on FOXO3a as elevated levels of this protein captured the effects of combined MEK and PARP inhibition.^52^

### Combined with immunotherapy

Despite its ground-breaking and paradigm-changing influence across various tumor types, immunotherapy is hampered by resistance mechanisms owing to the multiple layers of immune-evasive mechanisms used by tumors. The combination of immunotherapy with other anticancer agents has aimed to address this issue of resistance by simultaneously targeting multiple immune-evasive mechanisms. However, combination immunotherapy is in its infancy and consequently there are numerous outstanding questions remaining, including selection of the right drugs, optimization of treatment regimens, and management of toxicity.^55^

Unfortunately, in PDAC, immune therapy has not progressed as quickly as in other cancers as the PD-1/L1 axis inhibition has shown very limited activity.^56,57^ The only pancreatic tumors that have shown sensitivity to PD-1 inhibitors are those that are microsatellite-high (MSI-high), found only in a small minority of pancreatic cancers. In the rest of pancreatic cancers, PD-1 has had almost no evidence of activity.

PARP inhibition may induce sensitization to immunotherapy in the setting of *BRCA* mutations. The degree of tumor antigen burden has been associated with enhanced responses to immunotherapy.^58^ Therefore, increasing the neoantigen burden in immune-evasive cancers such as PDAC may help overcome resistance to immunotherapy. To this end, evidence suggests that PARP inhibition leads to increased tumor immunogenicity by increasing the tumor antigen burden as well as increasing PD-L1 expression in the tumor tissue.^59^

Furthermore, the immunotherapy-sensitizing effect of PARP inhibition was found to be dependent on the elevated PD-L1 levels.^60^ Further investigations suggest that this PD-L1 upregulation by PARP inhibition is mediated by the ATM/ATR/CHK1 pathway.^61^ Indeed, concomitant PD-1/PD-L1 inhibition with PARP inhibition should defend against the escape mechanism of increased feedback expression of checkpoint molecules.

Pembrolizumab (Keytruda) was the first therapeutic agent to be approved for use based solely on a biomarker—microsatellite instability-high/mismatch repair deficient (MSI-H/MMRd)—regardless of site of tumor origin. Indeed, it is reasonable to expect that defective DDR pathways other than MMR will lead to unique tumor-mutational signatures also associated with treatment response to immunotherapy.^62^ The tumor mutation burden (TMB) in germ line *BRCA1/2* mutant breast tumors is reported to be higher than in their *BRCA1/2-*proficient counterparts.^63^

Furthermore, LOH in ATM and MSI-H/MMRd has been found in gastric cancer patients and other DDR genes (*POLE, RAD51C, RAD17, POLD*) are often found in lung cancer and are associated with higher TMB and responses to immune checkpoint inhibitors (ICI).^64,65^ In addition to harboring high TMB, DDR defective-tumors share certain immune-related features. For example, tumors with BRCA1 or 2 mutations are associated with increased tumor-infiltrating lymphocytes, higher chemokine secretion, and increased expression of checkpoint molecules.^66^

Moreover, inhibition of PARP-insensitive tumors leads to increased DNA damage and genomic instability, consequently leading to apoptosis. It can be deduced that in cells that avoid apoptosis, the neoantigen burden will increase and subsequently lead to enhanced antitumor immune responses.^67^ Whether the above findings will mean enhanced efficacy for immunotherapy when combined with PARP inhibition for immune evasive tumors such as pancreatic cancer remains to be demonstrated.^55^

Other combination strategies for PARP inhibition and immunotherapy have been proposed to benefit patients with tumors without evidence of BRCA mutations or HRD phenotypes.^68^ Supported by pre-clinical evidence, a number of studies have begun to evaluate PARP inhibition plus ICI in various tumors. In PDAC, with the prior knowledge that platinum-sensitive tumors are associated with DDR deficiency, a phase II trial was begun, which is evaluating niraparib plus ipilimumab versus niraparib plus nivolumab in platinum-sensitive PDAC patients.^69^

Taken together, the above findings suggest that PARP inhibition potentially mobilizes antitumor immune responses, sensitizes PDAC to immunotherapy, and that the combination of ICI plus PARP inhibitor therapy is a promising strategy to overcome resistance in PDAC. Major published and ongoing trials with PARP inhibitors have been summarized elsewhere (Tables 1 and 2).

**Table 1.** Poly (ADP-Ribose) Polymerase Inhibitor Studies in Pancreatic Cancer

Agent	Patient population	Biomarkers	Study type	Combination	Results (ORR, DCR, PFS, OS, or SD)	Ref
Olaparib	Recurrent cancer (breast, ovarian, pancreatic, or prostate)	g*BRCA1/2* mutation	II	No	ORR 26% and 21.7% in patients with pancreatic cancer.	^27^
Veliparib	16 patients, 5 (31%) BRCA1, 11 (69%) BRCA2 gm. 14 (88%) received prior platinum-based therapy	g*BRCA1/2,* PALB2 mutation	II	No	PRs (6%) PD (25%) SD 11 (69%) PD as: median PFS 1.7 months, median OS 3.1 months (95% CI 1.9–4.1)	^28^
Olaparib	POLO study, 144 participants sensitive to platinum agents	gBRCA1/2 mutations	III	No	Improved Pfs (7.4 months olaparib vs. 3.8 months placebo).	^30^
ABT-888 (Veliparib)	SWOG S1513 trial mFOLFIRI+veliparib vs. FOLFIRI in second-line setting 143 participants, standard eligibility study	Germ line/somatic *BRCA1/2* mutations, and other DDR markers as correlates not necessary for enrolling	II	mFOLFIRI	Permanently closed before reach it end-point	^41^
Rucaparib	RUCAPANC trial: 19 BRCA+ relapsed mPAC pts	g*BRCA1/2* mutation	II	N/A	ORR was 11% (1 PR, and 1 CR); DCR ≥12 weeks was 32%	^29^
Veliparib	52 pts with BRCA+/PLAP2+	*BRCA1/2* mutation	I	Gemcitabine and cisplatin	52 pts with BRCA+/PLAP2+. Median OS 15.5 months triplet vs. 16.4 doublet	^43^
Olaparib	66 patients, nonselected for biomarkers	N/A	I	Gemcitabine 600 mg/m^2^	No significant difference	^64^
Olaparib	Pre-clinical, *in vitro* xenografts, 96 PC cells. Goal to assess ability of combination Chk1-PARP1 inhibition to sensitize to radiation therapy	N/A	I	Chk1 inhibitor AZD7762	AZD7762+olaparib is a significant radiosensitization in p53 mutant MPC	^65^

CR, complete response; DCR, disease control rate; DDR, DNA damage repair defect; MPC, metastatic pancreatic cancer; ORR, objective response rate; OS, overall survival; PARP, poly (ADP-ribose) polymerase; PFS, progression-free survival; PR, partial response; SD, stable disease.

**Table 2.** Ongoing Studies with Poly (ADP-Ribose) Polymerase Inhibitors

Clinical trial identifier	Agent	Study setting	Tumor site	Patient population	Single/combined	Status
NCT03337087	Rucaparib	Phase II	Metastatic pancreatic, colorectal, gastroesophageal, or biliary cancer	Third-line metastatic disease progressed in prior chemotherapy	Combined liposomal irinotecan, fluorouracil, leucovorin, calcium	Recruiting
NCT03601923	Niraparib	Phase II	Pancreatic cancer	Advanced pancreatic adenocarcinoma with positive DDR, progressed in prior chemotherapy	Single agent	Recruiting
NIRA-PANC-NCT03553004	Niraparib	Phase II	Pancreatic cancer with DDR	MPC with positive DDR after prior chemotherapy	Single agent	Recruiting
Parpvax-NCT03404960	Niraparib	Phase II	Pancreatic cancer with DDR	Maintenance after platinum chemotherapy in HRD-positive advanced pancreatic cancer	Combined with ipilimumab or nivolumab	Recruiting
NCT01286987	Talazoparib	Phase I	Pancreatic cancer with DDR	Advanced or recurrent solid tumors (breast cancer, ovarian cancer, Ewing sarcoma, small-cell lung cancer, prostate cancer, pancreatic cancer) with deleterious or pathogenic BRCA mutations	Single agent	Completed
LODESTARNCT04171700	Rucaparib	Phase II	Pancreatic cancer with DDR	Solid tumors with HRD positive	Single agent	Recruiting

HRD, homologous recombination deficiency.

## Conclusions

The current standard of therapy provides limited benefit for patients with PDAC due to limited responses. Consequently, identifying novel therapeutic strategies with tolerable toxicity for this patient population is of the utmost concern. To this end, PARP inhibition has emerged as a safe and effective treatment option with success being found in various tumors, including those originating from the ovaries, prostate, and breast.

Based on these findings, PARP inhibition has been studied in PDAC harboring *BRCA* mutations, and a recent landmark trial (POLO) demonstrated significant improvement in efficacy from the previous standard of treatment in this patient subpopulation. Future trials evaluating PARP inhibitors in PDAC tumors with non-*BRCA* DDR genes are warranted to expand this indication further beyond. In addition, combination strategies should garner attention as pre-clinical and clinical evidence suggests potential PARP inhibitor synergy with chemotherapy, immunotherapy, and other targeted therapies.

Our findings suggest an exciting transition period for the standard of care for metastatic PDAC with improvement in the clinical outcomes.

## Abbreviations Used

CR: complete response
DCR: disease control rate
DDR: DNA damage response
DSBs: double-strand breaks
HA: hyaluronic acid
HR: homologous recombination
ICI: immune checkpoint inhibitors
MEK: mitogen-activated protein kinase kinase
MPC: metastatic pancreatic cancer
MSI-H/MMRd: microsatellite instability-high/mismatch repair deficient
OR: objective response
ORR: objective response rate
OS: overall survival
PARP: poly (ADP-ribose) polymerase
PDAC: pancreatic ductal adenocarcinoma
PFS: progression-free survival
PR: partial response
SD: stable disease
SSB: single-strand break
TMB: tumor mutation burden
TME: tumor microenvironment
